# Cellular Stress-Modulating Drugs Can Potentially Be Identified by in Silico Screening with Connectivity Map (CMap)

**DOI:** 10.3390/ijms20225601

**Published:** 2019-11-09

**Authors:** Yurong Gao, Sungwoo Kim, Yun-Il Lee, Jaemin Lee

**Affiliations:** 1 Department of New Biology, Daegu Gyeongbuk Institute of Science and Technology (DGIST), Daegu 42988, Korea; gaoyuri212@dgist.ac.kr (Y.G.); sungwookim@dgist.ac.kr (S.K.); 2 Well Aging Research Center, Daegu Gyeongbuk Institute of Science and Technology (DGIST), Daegu 42988, Korea

**Keywords:** cellular stress, endoplasmic reticulum stress, ER stress, mitochondrial stress, oxidative stress, hypoxia, connectivity map, CMap, drug discovery

## Abstract

Accompanied by increased life span, aging-associated diseases, such as metabolic diseases and cancers, have become serious health threats. Recent studies have documented that aging-associated diseases are caused by prolonged cellular stresses such as endoplasmic reticulum (ER) stress, mitochondrial stress, and oxidative stress. Thus, ameliorating cellular stresses could be an effective approach to treat aging-associated diseases and, more importantly, to prevent such diseases from happening. However, cellular stresses and their molecular responses within the cell are typically mediated by a variety of factors encompassing different signaling pathways. Therefore, a target-based drug discovery method currently being used widely (reverse pharmacology) may not be adequate to uncover novel drugs targeting cellular stresses and related diseases. The connectivity map (CMap) is an online pharmacogenomic database cataloging gene expression data from cultured cells treated individually with various chemicals, including a variety of phytochemicals. Moreover, by querying through CMap, researchers may screen registered chemicals in silico and obtain the likelihood of drugs showing a similar gene expression profile with desired and chemopreventive conditions. Thus, CMap is an effective genome-based tool to discover novel chemopreventive drugs.

## 1. Introduction

Recent progresses in public health, the health care system, and medicine have greatly helped to extend our life span [1]. However, extended life span inevitably increases the risk of aging-associated diseases including cardiovascular diseases and cancers. Furthermore, a surplus of food consumption and lack of physical activity from a sedentary lifestyle has led to the drastic increase of obesity and its associated metabolic disorders such as type 2 diabetes [2,3]. Recent studies have demonstrated that aging-associated diseases, metabolic disorders, and cancers are caused by prolonged exposure to cellular stresses such as endoplasmic reticulum (ER) stress, mitochondrial stress, heat shock stress, and oxidative stress [4,5]. For example, the development of leptin resistance and insulin resistance leads to obesity and type 2 diabetes, respectively, and chronic inflammation and cellular stresses, including ER stress, oxidative stress, and mitochondrial stress have been reported to contribute to leptin and insulin resistance [4,5,6,7]. Furthermore, metabolic and cellular stresses also play a crucial role in the development of cancer and its pathophysiology [4,8,9]. Chronic exposure of cells to cellular stresses such as oxidative stress may lead to tumorigenesis; however, elevated cellular stresses such as hypoxia and ER stress may kill cancer cells [4,8,9]. Indeed, cancer cells have been shown to actively employ stress responses (e.g., unfolded protein response (UPR) against ER stress) to survive from excess cellular stresses [4,8,9]. Therefore, alleviating certain cellular stresses may prevent the development of cancer, whereas suppressing adaptive responses and escalating stresses can be useful in removing existing cancer cells [4,8,9].

**Figure 1:**
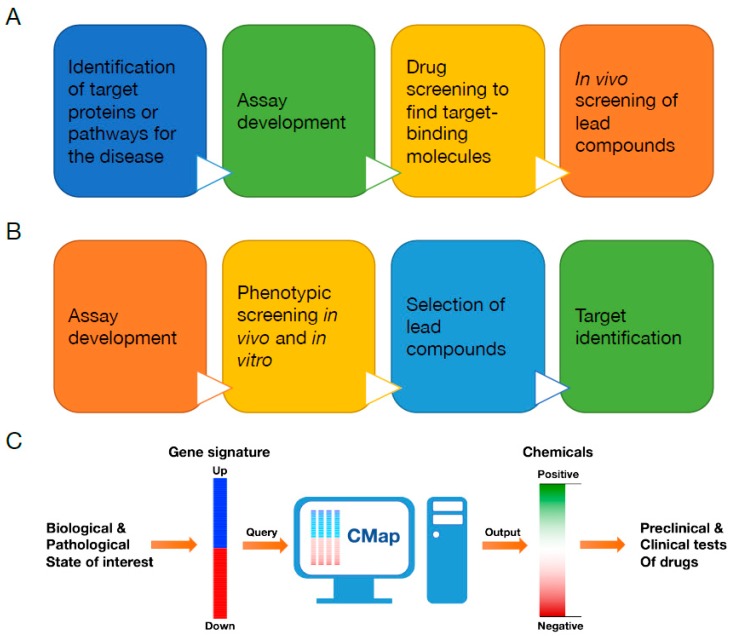
Drug discovery using the connectivity map (CMap). (**A**) Target-based drug discovery. (**B**) Phenotypic drug discovery. (**C**) CMap-based drug discovery. Gene signature of the biological or pathological state of interest can be used as a query to search through CMap. CMap provides the search result as a list of small molecules scored to predict their probability to mimic or reverse gene expression profiles of the state of interest. Candidate chemicals can be further tested in in vitro cell culture and in vivo animal experiments before proceeding with clinical trials to human subjects.

Therefore, developing chemopreventive ways to target appropriate cellular stresses could be an effective prevention and therapeutic treatment toward various aging-associated disorders [10]. However, cellular stresses and related molecular responses are mediated by a myriad of molecules encompassing multiple signaling pathways [4,5]. In addition, categorized cellular stresses do not take place solely inside of cells; instead, several stresses appear altogether [4,5]. For this reason, a target-based drug discovery process (reverse pharmacology) may not be adequate to discover novel chemicals that can address cellular stresses and associated disorders, although this is currently being used widely in academia and pharmaceutical companies (Figure 1A).

Phenotypic drug discovery (forward pharmacology) started to regain interest recently due to its potential usefulness in finding novel drugs to target complex diseases wherein the mechanism needs to be understood further, thanks to recent technological advances in cell-based phenotypic screening and analysis of vast genomic data (Figure 1B) [11]. The connectivity map (CMap) is an online genome-based database established by Todd R. Golub’s group at the Broad Institute (Boston, MA, USA), and catalogs transcriptome data from cultured cells treated individually with small molecules (Figure 1C) [12,13]. By searching on CMap, researchers can screen registered chemicals in silico and obtain the list of drugs displaying a similar gene expression profile with the desired biological or pathological conditions as a rank. Numerous studies have successfully demonstrated CMap’s potential as an effective pharmacogenomic drug discovery tool. In this article, we review the current understanding of cellular stresses and signaling responses, and discuss CMap as a potentially useful in silico drug screening tool to unearth novel drugs and phytochemicals to address cellular stresses and their related disorders.

## 2. Cellular Stresses

### 2.1. Heat Shock Stress and Heat Shock Response

Newly synthesized proteins form their native tertiary structure primarily based on their thermodynamic stability [14]. However, certain environmental conditions (e.g., heat, over-nutrition) and mutations within proteins often disturb proper protein folding and lead them to form aggregates [14]. Studies have shown that accumulated misfolded proteins and their aggregates cause many debilitating diseases, notably neurodegenerative diseases such as Alzheimer’s disease, Huntington’s disease, and Parkinson’s disease [15]. In order to facilitate appropriate protein folding and to prevent misfolded protein from forming aggregates, cells produce chaperone proteins such as cytoplasmic heat shock proteins (HSPs) and ER chaperones [15,16,17,18]. 

Heat shock response was initially reported from the observation in which active transcription (chromosomal puffs) was induced by heat treatment in the saliva gland of a fruit fly, *Drosophila busckii* [19]. Many of these loci have been identified to encode HSPs which are categorized and named based on their molecular weights—small HSPs, HSP40, HSP60, HSP70, HSP90, and HSP110 [20]. Although the specific role and mechanism of each HSP still needs to be investigated, HSPs generally function cytoprotectively [21,22,23]. One of the widely studied roles of HSPs is to function as molecular chaperones. They bind to misfolded and unfolded proteins, thus helping in folding and preventing them from forming aggregates [21,22,23]. Additionally, HSPs have been shown to modulate protein localization inside of cells and to promote antigen presentation [24]. 

**Figure 2:**
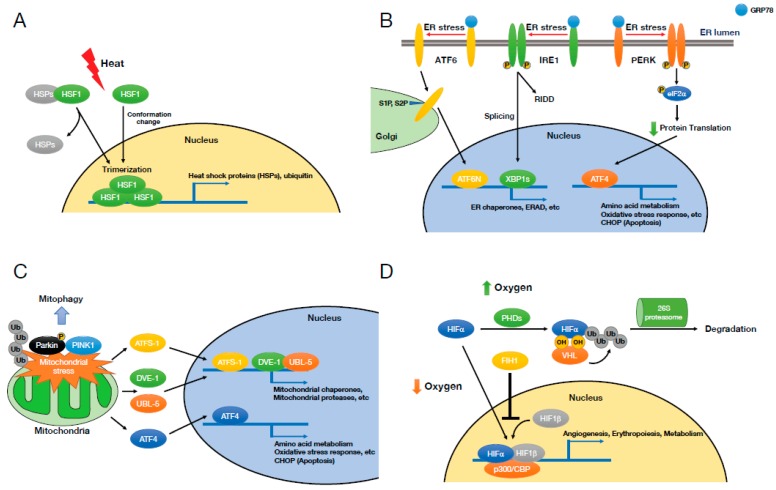
Cellular stresses and signaling responses. (**A**) Heat shock stress and heat shock response. The stressors such as heat lead to releasing of heat shock proteins (HSPs) from heat shock factor 1 (HSF1) or directly changing the conformation of HSF1 resulting in its trimerization, nuclear translocation, and target gene transcription. (**B**) Endoplasmic reticulum (ER) stress and unfolded protein response (UPR). The accumulation of unfolded or misfolded proteins activates three ER transmembrane proteins—activating transcription factor-6 (ATF6), inositol requiring protein-1 (IRE1), and protein kinase RNA-like ER kinase (PERK). ATF6 and IRE1 generate the functional transcription factors, ATF6N and spliced form of X-box binding protein 1 (XBP1s), which translocate to the nucleus and transcribe their target genes, whereas PERK suppresses protein translation and thus reduces protein load into the ER. (**C**) Mitochondrial stress and mitochondrial unfolded protein response (UPR^mt^). Mitochondrial stress activates several transcription factors, activating transcription factor associated with stress-1 (ATFS-1) and defective proventriculus (Drosophila) homolog-1/ubiquitin-like 5 (DVE-1/UBL-5) (*Caenorhabditis elegans*) and ATF4 (mammals), which promote their target gene expression to restore mitochondrial homeostasis. Mitochondrial stress also triggers autophagy (mitophagy) via Parkin and Pink1. (**D**) Hypoxia and hypoxia-induced factor. Under normoxia, hypoxia-inducible factor α (HIFα) is hydroxylated on proline by prolyl hydroxylase domain enzymes (PHDs) or on asparagine by factor inhibiting HIF1 (FIH1), and the activity of HIFα is suppressed by its von Hippel–Lindau (VHL)-mediated ubiquitylation and degradation or its loss of the interaction with p300/CREB-binding protein (CBP).

Heat shock response including HSP expression is induced not only by heat but also by other cellular stresses such as oxidative stress, osmotic stress, and exposure to heavy metals [21,22,23]. Subsequently, these stresses activate heat shock transcription factors (HSFs), a major transcription factor family mediating heat shock response. However, it is not understood clearly how HSFs sense cellular stresses. There are several isomers of HSFs (6 isoforms were identified in human—HSF1, HSF2, HSF4, HSF5, HSFX, and HSFY), and HSF1 is the most extensively studied among HSF isomers [22,25]. HSF1 exists as an inactive monomer in cytosol under normal conditions. In response to various stressors, HSF1 becomes an active transcription factor by forming a homotrimer and translocates to the nucleus [26,27,28]. Although the details of how the structure and activity of HSF1 are regulated are still under investigation, it has been suggested that physical interaction between HSPs and HSF1 leads to HSF1’s monomerization and cytosol localization under unstressed state, and in turn inhibits HSF1’s activity [29,30]. Under heat shock stress, HSPs are released from HSF1 probably by recruiting to unfolded or misfolded proteins, which subsequently allows HSF1 to form a homotrimer, to translocate to the nucleus, and to transcribe its target genes with unique HSF1 binding promoter (heat shock element) (Figure 2A) [22,25,26,27,28,29,30]. The changes in HSF1’s intrinsic structure itself during environmental stress, especially heat, have been shown to promote HSF1’s homotrimerization and nuclear translocation (Figure 2A) [31]. Furthermore, various post-translational modifications such as acetylation and phosphorylation have been shown to modulate HSF1’s activity [22,25].

Several studies have documented the role of heat shock response in aging, decreased and impaired function of HSF1 and other protein quality control machinery during aging have been reported, and further HSF1 activation was shown to increase the life span in a worm, *Caenorhabditis elegans* [22,32,33]. In addition, increased expression of HSF1 and HSP70 helps to ameliorate pathologies of neurodegenerative diseases such as Huntington’s disease, Parkinson’s disease, and amyotrophic lateral sclerosis (ALS) in mouse and fly models [34,35,36,37]. Moreover, mice deficient of HSF1 are resistant to form tumors under oncogenic conditions, suggesting that heat shock response protects tumor cells from cellular stresses and promotes their survival and proliferation [38].

### 2.2. Endoplasmic Reticulum (ER) Stress and Unfolded Protein Response (UPR)

The ER is an intracellular organelle that can be found in all eukaryotic cells. The ER bound with ribosomes (rough endoplasmic reticulum (RER)) is the major place to synthesize secretory and membrane proteins. The ER also produces lipids and stores intracellular calcium [5,39]. Newly translated proteins are moved into the ER lumen where they are folded into their native structure and also modified post-translationally by disulfide bond formation and glycosylation. Within the ER lumen, the quality control machinery such as ER chaperones helps to ensure proper protein folding [16]. However, when the ER fails to secure proper folding of ER proteins, protein homeostasis (proteostasis) is perturbed, and such a condition is referred to as ER stress [5,40]. Although the accumulation of unfolded or misfolded proteins beyond ER’s folding capacity is a primary cause of ER stress, metabolic stress, over-nutritional condition, and other cellular stresses also induce ER stress [5,40]. Under ER stress, cells employ an adaptive mechanism, UPR, to reestablish the ER homeostasis. The initial goal of the UPR signaling is to restore the ER proteostasis by increasing the expression of genes which promote protein folding and attenuating general protein translation which reduces additional protein load into the ER [5,40]. In addition, terminally misfolded proteins in the ER are translocated to the cytoplasm and degraded by the 26S proteasome, which is known as ER-associated degradation (ERAD) [41]. However, when ER proteostasis is not restored after these initial responses, UPR signaling launches cell death pathways [5,40].

The UPR signaling is initiated by three ER-located transmembrane proteins in metazoans, namely, inositol requiring protein-1 (IRE1), protein kinase RNA-like ER kinase (PERK), and activating transcription factor-6 (ATF6) (Figure 2B). IRE1 (yeast) or IRE1α (mammal) is a type I transmembrane protein residing in the ER and consists of an ER-lumenal domain and a cytoplasmic region with serine/threonine kinase domain and a ribonuclease (RNase) domain [42,43]. Under normal conditions, IRE1/IRE1α exists as a monomer by physical association of its ER-lumenal domain with an ER chaperone, glucose-regulated protein 78 kDa (GRP78). However, under ER stress which demands more ER chaperones to help the folding of unfolded or misfolded proteins, GRP78 is released from IRE1/IRE1α, which then triggers the dimerization/oligomerization of IRE1/IRE1α. Dimerization/oligomerization in turn leads to auto-transphosphorylation of IRE1/IRE1α at multiple sites including Ser724 of mammalian IRE1α [44,45], which ultimately activates the RNase domain of IRE1/IRE1α [46]. The RNase domain of IRE1/IRE1α selectively excises a 252-base intron of *HAC1* mRNA by IRE1 (yeast) and a 26-base fragment from *XBP1* (X-box binding protein 1) mRNA (*XBP1u*) by IRE1α (mammal) [47,48,49]. Spliced *HAC1* and *XBP1* (*XBP1s*) mRNA generate functional transcription factors, Hac1p and XBP1s protein, which translocate to the nucleus and transcribe their target genes which are generally involved in protein folding, ER biogenesis, and ERAD to restore ER proteostasis (Figure 2B) [47,48,49]. In addition to the splicing of HAC1 and XBP1 mRNA, IRE1/IRE1α cleaves and downregulates miRNAs, mRNAs, and other ER-associated RNAs, which is referred to as regulated IRE1-dependent decay (RIDD) [50,51,52,53]. XBP1s protein also shows various crosstalks with other signaling molecules including p38 MAPK, IKKβ, p85α/β, BRD7, PGC-1α, and FOXO1, which regulate XBP1s activity and its intracellular localization, and also modulate systemic glucose and lipid metabolism [54,55,56,57,58,59].

PERK is an ER-residing type I transmembrane protein composed of an ER-lumenal domain and a cytoplasmic serine/threonine kinase domain. The ER-lumenal domain of PERK is structurally homologous with the one of IRE1α, thus the dissociation of GRP78 from PERK monomer upon ER stress prompts homodimerization, auto-transphosphorylation, and activation of the kinase domain of PERK (Figure 2B) [60]. The activated PERK subsequently phosphorylates eukaryotic translation initiation factor 2 subunit alpha (eIF2α) at Ser51, resulting in the suppression of the assembly of ribosomal complex and global protein translation [61]. Despite the suppressed protein translation by eIF2α phosphorylation, certain transcription factors such as ATF4 and ATF5 can be actively translated due to multiple upstream open reading frames (uORFs) in their mRNA [62]. ATF4 then induces the expression of the proapoptotic transcription factor, C/EBP homologous protein (CHOP), which has been proposed as a major mediator of ER stress-induced apoptosis (Figure 2B) [63,64].

ATF6 is a type II ER transmembrane protein consisting with an ER-lumenal domain sensing the ER stress and a cytoplasmic domain that is a bZIP transcription factor [65]. The transcriptional activity of ATF6 remains inhibited without ER stress due to its retention in the ER via its physical association with GRP78. The dissociation of GRP78 from ATF6 under ER stress allows ATF6 to translocate to the Golgi where it is cleaved by site-1 protease (S1P) and S2P (Figure 2B) [66]. After cleavage, the cytoplasmic domain of ATF6 (N-terminal ATF6, ATF6N), an active transcription factor, translocates to the nucleus and transcribes its target genes in order to restore ER proteostasis (Figure 2B) [65].

ER stress and UPR signaling play a critical role in metabolic regulation and diseases [5]. Increased ER stress has been reported in several metabolically important tissues such as the liver, hypothalamus, and white adipose tissues of obese animal models [67,68]. Furthermore, the treatment of chemical chaperones alleviating ER stress, such as 4-phenylbutyric acid (4-PBA), and tauroursodeoxycholic acid (TUDCA) reduces ER stress and restores insulin and leptin sensitivity in animal models and human subjects, which suggests that modulating ER stress and its associated signaling pathways can be a useful therapeutic treatment to various metabolic diseases [67,69,70,71]. Additionally, several recent efforts have identified numerous novel chemicals as specific modulators of the individual UPR factors such as IRE1α, PERK, and ATF6 [72,73,74].

Cancer cells are constantly exposed to elevated ER stress and thus employ UPR and other signaling responses to ensure their survival from ER stress. Increased expression of UPR signaling factors such as XBP1s correlates with poor prognosis of several cancers such as glioblastoma, breast cancer and leukemia, and pharmacological or genetic inhibition of UPR responses demonstrates varying degrees of tumor-suppressing effects [75,76]. In addition, one of the mechanisms of Food and Drug Administration (FDA)-approved bortezomib, a proteasome inhibitor against multiple myeloma and mantle cell lymphoma, is to trigger ER stress-induced cell death in these cancer cells [77].

### 2.3. Mitochondrial Stress

Mitochondria are organelles derived from alphaproteobacteria that were engulfed by a eukaryotic progenitor before evolving as endosymbionts between 1 to 2 billion years ago [78,79]. Mitochondria form a highly dynamic network and continually undergo fusion and fission [80]. Mitochondria primarily function as a powerhouse of eukaryotic cells with oxidative phosphorylation protein complexes that are involved in electron transport and ATP synthesis. Mitochondria also perform crucial functions in many essential metabolisms and signaling pathways including iron–sulfur cluster synthesis, calcium buffering, and stress responses such as autophagy and apoptosis [7,81,82,83]. It is therefore not surprising that their dysfunction has been associated with a variety of diseases such as neurodegeneration, metabolic disease, heart failure, and cancer [7,81,82,83]. 

Eukaryotic cells have evolved multiple stress responses and adaptations to recognize and resolve mitochondrial dysfunctions. Protease-mediated mitochondrial protein quality control has been known for many years as the first line of defense against mitochondrial damage through the degradation of non-assembled proteins and misfolded proteins. The main ATP-dependent proteases performing protein surveillance are the Lon protease homologue (LONP), Clp protease proteolytic subunit (CLPP), intermembrane AAA protease (Yme1), and matrix AAA protease (AFG3L2/SPG7). Two ATP-independent proteases participate as well in mitochondrial protein quality control—mitochondrial inner membrane protease Atp23 homologue (ATP23) and intermembrane Ser protease (HTRA2) [84].

A recent series of studies has revealed that mitochondrial unfolded protein response (UPR^mt^) counteracts mitochondrial damage. Mitochondrial proteotoxic stress activates the UPR^mt^, which results in increased transcription of mitochondrial chaperones to help mitochondrial protein folding and proteases to degrade misfolded proteins. The mechanistic understanding of how the UPR^mt^ regulates the transcription has been extensively studied in *C. elegans*, in which the matrix protease CLPP digests unfolded or unassembled mitochondrial proteins into peptides. These peptides are transported to the cytoplasm and induce a transcriptional response in the nucleus via activating transcription factor associated with stress-1 (ATFS‑1). ATFS-1 is normally imported into mitochondria where it is degraded by the LONP. However, in response to mitochondrial stress, ATFS-1 accumulates in the cytosol and subsequently traffics to the nucleus (Figure 2C) [85]. In addition to ATFS-1, mitochondrial stress also induces ubiquitin-like 5 (UBL‑5) expression and UBL-5 protein forms a complex with defective proventriculus (Drosophila) homolog-1 (DVE-1), a transcription factor, which translocates into the nucleus (Figure 2C) [86]. In the nucleus, ATFS‑1 and DVE‑1-UBL‑5 induce the transcription of mitochondrial chaperones and proteases [85,86]. However, in mammals, the understanding of UPR^mt^ is not clear. It has been reported that the transcription factor ATF5 regulates a mammalian UPR^mt^ and appears to function as mammalian orthologs of ATFS-1 [87]. Another study found that mammalian UPR^mt^ altered the expression of nuclear genes including mitochondrial chaperonins that is involved in protein folding, concurrently with reduced protein synthesis in the matrix via rapid but reversible translational inhibition. Functional studies also revealed that transcriptional repression and LON protease-mediated degradation of mitochondrial pre-RNA processing nuclease MRPP3 lead to defects in pre-RNA processing within the mitochondria, which in turn suppresses the translation of mtDNA-encoded proteins, thereby reducing protein folding load in the mitochondrial matrix [88]. Another study demonstrated that ATF4 is a main player in the mitochondrial stress response in mammals, which acts downstream of the integrated stress response (Figure 2C). ATF4 promotes the expression of various cytoprotective genes, some of which reprogram cellular metabolism toward the synthesis of key metabolites, especially serine. Newly produced serine may promote lipid and phospholipid synthesis which have been known to be critical in mitochondrial stress [89]. Moreover, UPR^mt^ attenuates mitochondrial translation by decreasing the levels of mitochondrial ribosomal proteins independently of ATF4 [89].

Mitophagy is selective autophagy which degrades damaged mitochondria, thereby maintaining a healthy mitochondrial population. Mitophagy requires PINK1, a kinase that is imported into mitochondria under normal conditions and subsequently degraded by proteolysis. When mitochondria are depolarized and dysfunctional, PINK1 is stabilized on the outer mitochondrial membrane, and recruits Parkin, a ubiquitin ligase, on the damaged mitochondria. The outer membrane on the mitochondria is then ubiquitylated by Parkin. Consequently, the poly-ubiquitinated mitochondria are selectively recognized and bound by autophagy machinery, triggering the selected degradation of mitochondria (Figure 2C) [90,91].

Aging accompanies the accumulation of dysfunctional mitochondria and mutations in genes involved in mitochondrial function, which affect life span [92,93]. In addition, mitochondrial dysfunction is linked to various metabolic diseases such as obesity, type 2 diabetes, hypertension, and non-alcoholic fatty liver disease [83,94,95]. Moreover, as exemplified by Parkinson’s disease, impaired mitophagy and mitochondrial dysfunction have been suggested to cause various neurodegenerative diseases [96,97]. Mutations in PINK1, PARK2 (Parkin), ATP13A2, and DJ-1 impair mitophagy and elicit mitochondrial dysfunction, in turn leading to autosomal recessive Parkinson’s disease [98,99]. Additionally, mitochondrial dysfunction is observed in Alzheimer’s disease, Huntington’s disease, ALS, and other neuropathies, but its causal role in these neurodegenerative diseases has not yet been established [96,100]. Since Otto Warburg discovered that cancer cells mainly utilize aerobic glycolysis to produce lactate from glucose in the presence of oxygen (Warburg effect), the defects of oxidative phosphorylation in mitochondria was believed to produce this Warburg effect in cancer cells [101]. However, recent studies document that cancers alter the mitochondrial function instead of inactivating it to produce metabolite needed by cancer cells [101,102]. Mutations in tricarboxylic acid (TCA) cycle enzymes such as isocitrate dehydrogenase 2 (IDH2), succinate dehydrogenase, and fumarate hydratase are frequently found in human cancers [101,102], and the inhibitor of mutant IDH2, enasidenib, was approved by the FDA to treat acute myeloid leukemia [103,104].

### 2.4. Hypoxia

Molecular oxygen (O_2_) is a critical substrate for mitochondrial ATP production, signaling, and numerous cellular metabolisms. The maintenance of O_2_ homeostasis is, therefore, essential for the development of multicellular animal life. O_2_ deprivation (hypoxia) is the condition in which cellular O_2_ delivery does not meet the demand. Hypoxia is one of defining features of solid tumors associated with increased therapeutic resistance [105,106,107]. The central mediators of cellular adaptation to hypoxia are hypoxia-inducible factors (HIFs), a family of heterodimeric basic helix-loop-helix transcription factors composed of an oxygen-sensitive HIFα subunit and a constitutively expressed HIF1β subunit. Three HIFα subunits are identified in mammals—HIF1α, HIF2α, and HIF3α. In the presence of oxygen, HIFα subunits are rapidly hydroxylated on proline residues by a group of prolyl hydroxylase domain (PHD) enzymes. Once hydroxylated, HIFα binds to the von Hippel–Lindau (VHL) protein, an E3 ubiquitin ligase targeting HIFα for proteasomal degradation (Figure 2D). In another mode of HIFα regulation, HIFα undergoes asparaginyl hydroxylation by factor inhibiting HIF1 (FIH1), which inactivates HIFα transcriptional activity by preventing its interaction with the transcriptional co-activator CREB-binding protein (CBP) and histone acetyltransferase p300 (Figure 2D). Thus, PHDs and FIH1 function as O_2_-dependent oxygenases to post-translationally modify HIFs to suppress their transcriptional activity [108]. Conversely, during hypoxia, PHD and FIH activity is suppressed, resulting in HIFα stabilization and dimerization with HIF1β. Subsequently, the HIF dimer translocates to the nucleus and transcribes its target genes with hypoxia-responsive elements (HREs), HIF-binding promoters (Figure 2D) [109,110,111]. HIF target genes generally stimulate vascularization (VEGF), raise the blood’s oxygen carrying capacity (erythropoietin), and modulate mitochondrial metabolism.

The largest group of genes regulated by HIF1 are associated with glucose metabolism. HIF1 can increase the rate of glucose uptake through the upregulation of the glucose transporters, GLUT1 and GLUT3. Furthermore, HIF1 stimulates enzymes responsible for the glycolytic breakdown of intracellular glucose to pyruvate. HIF1 also upregulates lactate dehydrogenase A (LDHA) which converts pyruvate to lactate. The lactate can then be transported out of the cell through the action of the HIF-inducible cell surface monocarboxylate transporter 4 (MCT4) [112,113]. Thus, HIF1 activation leads to an increase in glycolysis. 

Mitochondria and O_2_ are inextricably intertwined. There are several mechanisms by which HIF signaling can affect mitochondrial function. HIF1 induces the expression of pyruvate dehydrogenase kinase 1 (PDK1), which phosphorylates and inactivates the mitochondrial pyruvate dehydrogenase (PDH) and blocks the conversion of pyruvate to acetyl-CoA, thereby suppressing the TCA cycle and attenuating oxidative phosphorylation and excessive toxic reactive oxygen species (ROS) production [114]. HIF1 also modulates mitochondrial metabolism by replacing the cytochrome c oxidase subunit COX4‑1 with COX4‑2, in which HIF1 increases the transcription of COX4-2 while downregulating COX4-1 protein levels by augmenting the expression of Lon protease. COX4-2 is more efficient at facilitating the electron transfer to O_2_, and thereby lowers ROS levels [115]. In addition, HIF1 upregulates BNIP3 and BNIP3L, which promote mitophagy [105]. In another report, the researchers suggest chronic hypoxia could be used as an unexpected treatment for defects in the mitochondrial respiratory chain [116]. 

Regulation of hypoxic responses via the HIFs is well established, but growing evidence also indicates that HIF-independent mechanisms are also involved. In one study, hypoxic response depends on the accumulation of lactate which binds to the NDRG3 protein and stabilizes it. NDRG3 is an oxygen-regulated protein and also a substrate of the PHD2/VHL system. The stabilized NDRG3 mediates hypoxia-induced activation of the Raf-ERK pathway and promotes angiogenesis and cell growth [110]. In another study, hypoxia promotes survival of in vitro and in vivo models of Friedreich’s ataxia by restoring the steady-state levels of Fe–S clusters independently of HIF. Mitochondrial protein frataxin (FXN) participates in the biosynthesis of Fe–S clusters, and FXN-deficient yeast, human cells, and *C. elegans*, which cannot survive under normoxia, were able to grow continuously in ambient 1% O_2_, a hypoxic condition. This indicates that hypoxia somehow could directly promote Fe–S synthesis bypassing the requirement of FXN [117].

Cancer cells are constantly exposed to the hypoxic condition, thus cancer cells frequently employ various responses ameliorating hypoxic stress. Increased expression of HIF1α and HIF2α correlates with negative outcome of human tumors, and HIFs in cancer cells promote glucose metabolism and angiogenesis to help tumor proliferation and survival [118]. In addition to cancer metabolism, hypoxia signaling contributes to systemic glucose and lipid metabolism; depletion of HIF1α in pancreas β-cells causes glucose intolerance due to impaired insulin secretion [119]. Additionally, genetic and pharmacological suppression of HIF2α activity in the intestine alleviates hepatic steatosis of obese mice, whereas activation of HIF2α in the liver improves glucose metabolism and ameliorates type 2 diabetes [120,121,122].

### 2.5. Oxidative Stress 

Oxidation–reduction (redox) homeostasis is crucial to maintaining nearly all principal cellular processes. During the redox reaction, various oxidants and antioxidants are generated endogenously, and when oxidants are produced or obtained beyond the balancing redox capacity of cells, it leads to oxidative stress. ROS, which causes oxidative stress, includes not only narrowly defined ROS but also various other kinds of chemicals such as reactive nitrogen species, reactive chlorine/bromine species, reactive sulfur species, reactive carbonyl species, and reactive selenium species [123,124]. ROS is continuously generated during metabolism, which has been considered to facilitate accumulated DNA damages and ultimately lead to the development of cancers and cellular aging. ROS and accompanying oxidative stress also have been demonstrated to contribute to the pathophysiologies of various chronic diseases such as cardiovascular diseases, obesity, diabetes, and neurodegenerative diseases [125]. However, recent studies also show that ROS plays a beneficial role in many cellular functions. For example, ROS generated from phagocytes constitutes a pathogen-killing mechanism during phagocytosis. Furthermore, some ROS such as hydrogen peroxide (H_2_O_2_) and nitric oxide (NO) play a role in cellular signaling and in post-translational modifications of proteins such as sulfenylation and S-nitrosylation [126,127,128,129,130]. The majority of ROS is produced from the electron transport chain of mitochondria as a superoxide anion radical, O_2_•^−^, and most of O_2_•^−^ is converted to H_2_O_2_ by manganese superoxide dismutase (MnSOD) [131,132,133]. Additionally, NADPH oxidases, which are activated by growth factors, also generate H_2_O_2_ [134], whereas NO synthases produce NO [135].

Because oxidative stress can be produced at every cellular metabolic process, a myriad of signaling responses even in other stress responses are employed to curb oxidative stresses, which include NRF2-KEAP1, p53, MAPKs (JNK, p38 MAPK, ERK), PI3K/Akt, NF-κB, heat shock response, and UPR [125,136]. In general, the majority of these pathways exercise pro-survival responses, whereas some responses from JNK, p38, p53, and UPR pathways (e.g., CHOP) exert cell death [5,125]. Among these oxidative stress responses, NRF2-KEAP1 is regarded as one of the main regulators of the cellular antioxidant responses. NRF2 is a transcription factor and its protein levels are maintained at low under unstressed conditions by three E3 ubiquitin ligase complexes—KEAP1-CUL3-RBX1, β-TrCP-SKP1-CUL1-RBX1, and HRD1 [137]. However, KEAP1-CUL3-RBX1 is considered as a principal negative regulator responding to the changes of redox condition [136,137]. KEAP1 is a substrate adaptor protein of the CUL3-RBX1 E3 ligase complex and binds to NRF2 to prompt NRF2 ubiquitylation and its subsequent degradation during unstressed conditions. Under oxidative stress condition, excessive ROS reacts with cysteines (especially Cys151) at the N-terminal part of KEAP1, leading to its conformational changes and subsequent loss of affinity to NRF2. In turn, NRF2 translocates to the nucleus, forms a heterodimer with sMAF, and then transcribes its target genes with antioxidant response element, many of which contribute to antioxidant responses (e.g., glucose 6-phosphate dehydrogenase, 6-phosphogluconate dehydrogenase, malic enzyme 1, and isocitrate dehydrogenase 1, which are involved in NADPH production) [136,137].

Many studies have suggested that ROS and oxidative stress contribute to cellular senescence, aging, and aging-associated diseases [125]. During the progression of type 2 diabetes, pancreatic β-cell dysfunction is caused by increased ER stress, mitochondrial stress, and oxidative stress [138]. Moreover, ROS-induced DNA damage, in addition to the chemical modifications of macromolecules such as lipids and proteins, is considered to lead to the development of cancer, whereas many chemotherapy and radiation therapy treatments induce excessive oxidative stress to kill cancer cells [139,140]. Furthermore, genes in the KEAP1-NRF2 pathway are frequently mutated in certain cancers such as squamous cell carcinoma and lung adenocarcinoma showing the strong resistance to chemotherapy and radiation therapy [141,142].

## 3. Connectivity Map (CMap)

As we explained above, cellular stresses and their related signaling responses are mediated by various environmental factors (e.g., heat, oxidants, osmotic stress, and over-nutrition) and a plethora of signaling molecules. Therefore, finding chemopreventive and even therapeutic chemicals targeting cellular stresses and their associated diseases is challenging and has proven to be difficult with target-based drug discovery. Because of its potential advantage to address complex diseases that require more understanding of their mechanisms, and also recent advances in screening methods for phenotypic drug discovery such as cell-based phenotypic screening and pharmacogenomic analysis, phenotypic drug discovery has started to regain its interest and usage in drug screening.

Recently, the connectivity map (CMap) and the upgraded CMap (L1000) have been demonstrated as useful in silico drug screening tools to target cellular stresses and their related disorders [12,13,143,144]. The CMap (https://portals.broadinstitute.org/cmap/) is a gene expression compendium archiving gene expression data from cultured cells, treated with individual chemical perturbagens and whose ≈22,000 gene expression levels were analyzed with microarray (CMap Build 2 stores results from over 1300 chemical treatments that include a variety of phytochemicals).

Importantly, researchers can query the CMap with their gene expression signatures that consist of a list of genes upregulated and downregulated in the biological or pathological states of interest (Figure 1C). In order to compare the query signature to the entire microarray data in the CMap, which were generated from different cell lines and batches as well as with various doses and treatment time, CMap uses a nonparametric, rank-based pattern-matching analysis. In the end, CMap presents its query result as a list of drugs with a “connectivity score” ranging from +1 (positive connectivity) to -1 (negative connectivity) (Figure 1C). Drugs with a positive connectivity score may generate similar gene expression outcomes with the state of interest (query state), whereas ones with a negative score produce reverse gene expression patterns with the query. Additionally, drugs with a near zero score are unlikely to induce any related responses with the query state. Therefore, the CMap potentially provides a list of candidate chemicals which may mimic or reverse the biological or pathological state of interest [12,13].

Recently, increasing numbers of studies have used CMap to uncover promising small molecules to address various diseases. For example, searching on CMap with gene expression data from tissues (liver and hypothalamus) showing diminished ER stress and improved leptin/insulin receptor signaling as query signatures successfully identified celastrol as an effective leptin sensitizer and chemical chaperone ameliorating obesity in the leptin-resistant mouse model [145]. Celastrol is a phytochemical originally extracted from the root of the thunder god vine, *Tripterygium wilfordii*, which has been used as a medicinal plant in China and other East Asian countries as a treatment of inflammatory diseases such as rheumatoid arthritis [145,146]. Furthermore, using the gene expression signature of celastrol as a query on CMap uncovered that withaferin A is also a chemical chaperone and a leptin sensitizer, and significantly ameliorates obesity [147]. Similar to celastrol, withaferin A is also a phytochemical originally extracted from leaves, berries, and roots of *Withania somnifera*, a winter cherry (also called Ashwagandha in India), which has been used as a medicinal plant in India as a treatment of various disorders including inflammation, autoimmune diseases, tumors, stress, anxiety, and aging [148,149,150]. Besides the discovery of celastrol and withaferin A as chemical chaperones and anti-obesity drugs, CMap has been successfully utilized to uncover numerous chemicals with potentials to treat various other diseases, for example, COX2- and ADRA2A-targeting chemicals to treat type 1 and type 2 diabetes [151], tomatidine (a phytochemical from tomato plants) for skeletal muscle atrophy [152], anisomycin for spinal muscle atrophy [153], topiramate for inflammatory bowel disease [154], kaempferol (a phytochemical) for cigarette smoke-induced inflammation [155], pyrvinium for obesity [156], cannabidiol (a phytochemical from *Cannabis sativa*, marijuana plant) for diabetic cardiomyopathy [157], piperazine for central nervous system injury [158], and many other chemicals for cancers such as medulloblastoma [159], breast cancers [160,161], lung cancers [162,163], glioblastoma [164], ovarian cancer [165], prostate cancers [166], myeloma [167], atypical meningioma [168], leukemia [169,170,171,172], and many others [173,174,175].

Despite CMap’s promising potential as a genome-based and phenotypic drug discovery tool, CMap has limitations. First, constructing and expanding reference profiles of CMap database are time consuming and expensive because CMap is built upon full transcriptome analysis. Second, CMap is still built on data from limited numbers of small molecules and cell lines. In addition, gene expression data from cultured cells may not be appropriate to address the diseases happening in our body or in specific organs. Third, CMap results may not provide enough information about direct drug targets because CMap is a phenotypic drug discovery tool. To overcome these shortcomings, the same team at the Broad Institute who created the original CMap has developed the “next generation connectivity map” or L1000 (https://clue.io/) as part of the National Institutes of Health (NIH) Library of Integrated Network-Based Cellular Signatures (LINCS) initiative [144]. The current L1000 expands the original CMap by using nearly 28,000 perturbagens including over 19,000 small molecules and ≈7000 genetic modulations using knockdown with shRNAs and over-expression with cDNAs. L1000 also includes more cell lines (nine core cell lines) to test perturbagens or uses 3 to 77 variable cell lines for chemicals without characterized mode of action. Moreover, in order to build the new CMap through high-throughput screening at lower costs, L1000 uses only 1000 landmark transcripts as references instead of the full transcriptome, which the authors claim addresses ≈80% of the information in the entire transcriptome [144]. Collectively, L1000 alongside with the original CMap provide powerful in silico pharmacogenomic ways for researchers to discover novel small molecules targeting various diseases that currently do not have effective therapeutics due to their complicated pathophysiologies.

## 4. Conclusions and Future Perspectives

As our modern society enters the state of population aging, aging-associated diseases such as cardiovascular diseases, obesity, diabetes, neurodegenerative diseases, and cancers have become a major health threat as well as a serious economic and social burden. Even though tremendous efforts have expanded our understanding of the pathophysiologies of these disorders and have also developed numerous medications against them, such efforts still fell short to alleviate significantly chronic and aging-associated disorders. This is partly due to their compounding nature in which a myriad of genetic and environmental factors are interwoven with each other. Recently, a growing number of studies have documented that cellular stresses caused by the disruption of homeostasis within the cell contribute to the development of aging-associated diseases and have suggested that ameliorating these cellular stresses could be an effective prevention and therapeutic treatment. There are several uniquely categorized cellular stresses such as heat shock stress, ER stress, mitochondrial stress, oxidative stress, and hypoxia. However, it should be noted that cellular stresses do not occur individually but frequently happen together. Due to these complexities, developing chemopreventive and therapeutic treatments against cellular stresses and their associated diseases has not yet achieved any significant progress. However, recent technological and genomic advances bring new opportunities to tackle many debilitating chronic disorders. Among them, CMap and its upgraded L1000 are potentially powerful genome-based in silico drug discovery methods based on phenotypic drug discovery, and many studies have successfully used CMap to uncover novel chemicals to alleviate cellular stresses and aging-associated diseases. Furthermore, there are still many possibilities to expand CMap and L1000 in the future in order to be more effective as follows. (1) The numbers of perturbagens (small chemicals and genetic modulations) in CMap/L1000 could increase further, including numerous phytochemicals available currently, and CRISPR/cas9 could be also utilized as genetic perturbation. (2) CMap/L1000 could include more cell types, especially induced pluripotent stem cells (iPSC) and tissue-specific organoids. Additionally, (3) future CMap/L1000 or other pharmacogenomic tools could include more phenotypic information, including proteomic and epigenetic data and also high-content imaging profiles. Furthermore, (4) recent advances in machine learning could empower future genome-based in silico drug discovery tools by potentially providing the information about probable modes of action and target proteins of small molecules.

Historically, phytochemicals have provided huge medical benefits to humankind, as famously shown by salicin (from the willow tree and modified to aspirin®), morphine (from the opium poppy), cocaine (from coca leaves), guanidine (from the French lilac and modified to metformin), and many other examples. However, many phytochemicals’ potential medical benefits are still unknown. However, with recent phenotypic drug discovery tools such as CMap, we can finally be in the position to uncover novel functions of phytochemicals which could be both chemopreventive and therapeutic toward many chronic diseases caused by cellular stresses. 

## Abbreviations

β-TrCP	Beta-transducin repeat containing E3 ubiquitin protein ligase
AAA	ATPases associated with diverse cellular activities
AFG3L2	AFG3-like protein 2
ALS	Amyotrophic lateral sclerosis
AMPK	AMP-activated protein kinase
BNIP3L	BCL2 interacting protein 3 like
BRD7	Bromodomain-containing protein 7
CLPP	Clp protease proteolytic subunit
CUL1	Cullin 1
CUL3	Cullin 3
ERK	Extracellular signal-regulated kinase
FDA	Food and Drug Administration, USA
HRD1	HMG-CoA reductase degradation 1 homolog
IDH2	Isocitrate dehydrogenase 2
IKKβ	Inhibitor of nuclear factor kappa-B kinase subunit beta
JNK	c-Jun N-terminal kinase
KEAP1	Kelch-like ECH-associated protein 1
MAPK	Mitogen-activated protein kinase
NADPH	Nicotinamide adenine dinucleotide phosphate, reduced form
NF-κB	Nuclear factor kappa-light-chain-enhancer of activated B cells
NRF2	Nuclear factor erythroid 2-related factor 2
PGC-1α	Peroxisome proliferator-activated receptor gamma coactivator-1 alpha
PI3K	Phosphoinositide 3-kinase
PINK1	PTEN-induced kinase 1
RBX1	Ring box 1
SKP1	S-phase kinase-associated protein 1
sMAF	Small musculoaponeurotic fibrosarcoma
SPG7	Spastic paraplegia 7
TCA	Tricarboxylic acid
VEGF-B	Vascular endothelial growth factor B
